# Incorporating Modern Fault Ride-Through Standards into the Short-Circuit Calculation of Distribution Networks

**DOI:** 10.3390/s23218868

**Published:** 2023-10-31

**Authors:** Evangelos E. Pompodakis, Yiannis Katsigiannis, Emmanuel S. Karapidakis

**Affiliations:** 1 Institute of Energy, Environment and Climatic Change, Hellenic Mediterranean University, 71004 Heraklion, Greece; 2 School of Engineering, Power Systems and Energy Engineering, Hellenic Mediterranean University, 71004 Heraklion, Greece; katsigiannis@hmu.gr (Y.K.); karapidakis@hmu.gr (E.S.K.)

**Keywords:** distribution networks, fault analysis, fault ride-through, FRT standards, fuse melting time, relay tripping time, short-circuit calculation

## Abstract

Modern fault ride-through (FRT) standards in many countries require distributed generators to remain connected for a specified period during the fault by providing reactive current, to support voltage and prevent a massive renewable outage. As a result, short-circuit current is not constant, but it varies depending on the current and disconnection order of distributed generators (DGs). This time-varying short-circuit current complicates the estimation of the time it will take for an overcurrent relay or fuse to trip. The existing short-circuit calculation algorithms usually assume that the fault current is constant throughout the whole period of fault. This assumption may result in incorrect conclusions regarding the tripping time of protective devices in networks with high renewable penetration. This paper incorporates modern FRT standards into the fault analysis by considering the influence of fault current variations on the protective devices (relays, fuses), significantly increasing the accuracy of the estimated tripping time. Simulations carried out in a 13-bus and the IEEE 8500-node network indicate that the traditional short-circuit calculation approaches may miscalculate the tripping time of protective devices, with deviations up to 80 s, when applied to networks complying with modern FRT standards.

## 1. Introduction

### 1.1. Motivation and Challenges

Short-circuit calculation (SCC) is a fundamental application of modern distribution management systems (DMSs). Specifically, SCC is executed either on a cyclic basis or following a request for topology change, assuming all possible fault locations, in order to check for potential violations of circuit breaker/fuse breaking capability, the adequacy of the relay sensitivity, and the levels of earth fault currents [1]. It is employed to identify fault locations and to perform checks on the protection system, possibly involving an update of relay settings, prior to implementing feeder reconfiguration. Moreover, it constitutes a basic tool for several other online applications such as fault location, isolation, and supply restoration [1,2]. Therefore, SCC tools should be characterized by high accuracy to accomplish the aforementioned functionalities.

Modern standards require DGs to remain connected for a specified period during the fault, by injecting positive-sequence reactive current, depending on their point of common coupling (PCC) voltage [3,4]. Due to the different disconnection times of DGs, the fault current is no longer constant throughout the fault, but it varies, affecting the operation of protective devices. Therefore, SCC tools should be revised accordingly to accommodate the attributes of modern fault ride-through (FRT) standards as well as all the factors that affect the fault current, such as the disconnection order of DGs.

### 1.2. Literature Review

Currently, SCC is performed using time-domain tools [5,6,7,8] such as Simulink or steady-state solvers [9,10,11,12,13,14,15,16,17,18,19,20,21,22,23]. The former require a detailed representation of the system and dynamic response of DGs. As a result, they are very time consuming, and their applicability is restricted only to unrealistically small networks [4,5,6,7]. The latter compute the steady state of fault current, and thus, their computation time is significantly lower, enabling their real-time applicability in DMS applications of real networks.

Steady-state solvers are categorized depending on their computation time, coordinates (phase or sequence), modeling of DGs, and loads. In [9], an SCC method is introduced, in phase coordinates, according to which loads and fault are incorporated as constant impedances into the ***Y_BUS_*** matrix of the network. The method involves all the buses of the network, even the load buses, and thus the computation time is increased, especially in large networks. In [10], a fast SCC approach is formulated, in sequence components, using the superposition of the known pre-fault state and the state of a reduced-size post-fault circuit. In [11], two explicit matrices (named bus injection to branch current and branch current to bus voltage) are developed by utilizing the radial characteristic of distribution systems to calculate the variations in bus voltages, bus-current injections, and branch currents under faulted conditions in an efficient manner. In all the methods above [9,10,11], the authors completely neglect DGs, or alternatively, they assume they are immediately disconnected after the fault (no FRT), without contributing to the fault current.

In [12], the authors extended their previous work in [9] by incorporating DGs into the SCC. The short-circuit current contribution by the DGs is limited to the short-circuit current capacity of the switching devices by operating the inverter in a constant current mode. In [13], SCC is solved using superposition theory in sequence components, in a similar formulation as [10], accounting also for DGs. To expedite the computation time for short-circuit analysis, the problem is reformulated as a linear equation problem, which can then be efficiently resolved using the simple generalized minimal residual (SGMRES) method. In [14] a novel SCC approach is introduced in unbalanced microgrid systems, employing graph theory and a complex short-circuit MVA representation. Unlike the conventional Z_BUS_ method and the use of an inverse Y_BUS_ matrix with the lower and upper triangular matrix (LU) method, ref. [14] is based on the branch-path incidence matrix and a newly defined augmented incidence matrix. In [15], the authors consider the influence of environmental conditions on the calculation of DG fault currents. In [16], a generalized Δ-circuit is proposed, in sequence components, which enables the precise simulation of DGs. Specifically, the faulted system state is calculated by superimposing the pre-fault state with the Δ-circuit. The Δ-circuit includes only the active buses (namely DG and faulted buses and not the load buses), reducing the computation time considerably. The model of [16] is extended in [17] to islanded networks with virtual impedance current limiters. In fact, in reference [17], a modification is made to the formulation, disregarding the effect of a slack bus on fault currents, as is often the case in islanded microgrids. In [18], a whole-line fault analysis method for distribution networks with DGs is proposed, enabling the SCC in faults occurring not only at the location of network buses but also along the lines. In [19], the blinding effects caused on the overcurrent relay of a German low-voltage (LV) feeder are studied, using a tool for stationary short-circuit current calculations, based on the current-source superposition method. Finally, in [20], the authors study all the problems arising from the connection of DGs to distribution networks, such as blinding, islanding, and miscoordination. For their analysis, they apply the superposition rule, ignoring the loads prior to the short-circuit, that is, they simplistically assume a flat pre-fault voltage profile, while the DG currents are superimposed on the flat voltage profile using the network impedance matrix.

Sophisticated methods focusing on the modeling of particular types of DGs under fault conditions, such as inverter-based DGs [21], DGFIs [22], and HVDC-connected offshore wind farms [23], have recently been presented in the literature, as well. Specifically, in [21], authors study the existence of multiple equilibrium points in networks supplied by both grid-forming and grid-following DGs, depending on whether the grid-forming DG has reached saturation. In [22], an SCC method is presented, with an advanced modeling of the non-linear crowbar operation, under fault conditions, in double-fed induction generators (DFIGs). Finally, in [23], the FRT of offshore DFIGs connected to a bipolar high-voltage DC (HVDC) link is studied, considering faults occurring at one of the poles of the bipolar HVDC link.

All the aforementioned SCC methods assume that either all DGs remain connected, injecting a constant current throughout the whole period of the fault, such as in [12,13,14,15,16,17,18,19,20,21,22,23], or they are disconnected once the fault occurs, as in [9,10,11]. Therefore, the short-circuit current is assumed constant, examining only one time instant (snapshot) of the fault. Nevertheless, as already explained, modern FRT codes require DGs to remain connected for a length of time depending on their positive-sequence PCC voltage. Since DGs are connected to different locations, they experience different voltages and thus different disconnection times, causing the short-circuit current to be varied during the fault. As the DG penetration keeps growing, existing SCC methods [9,10,11,12,13,14,15,16,17,18,19,20,21,22,23] may lead to incorrect estimation regarding the sensitivity and tripping time of protective devices due to their assumption of a constant fault current profile. To the best of our knowledge, only one paper exists in the literature that incorporates into the SCC the disconnection order and time-varying current of DGs [24]. Specifically, Hooshyar et al. [24] propose an SCC model by considering the disconnection order of DGs, depending on their terminal voltages. DGs with terminal voltages below 0.5 pu will be disconnected in less than six cycles, while for higher voltages, it may take up to 120 cycles. The authors found that such a voltage-dependent disconnection order of DGs greatly affects the short-circuit current profile in networks with high DG penetration, and consequently, the tripping time of protection relays (for more details, refer to section IV of [24]).

### 1.3. Contributions of the Paper

**Figure 5:**
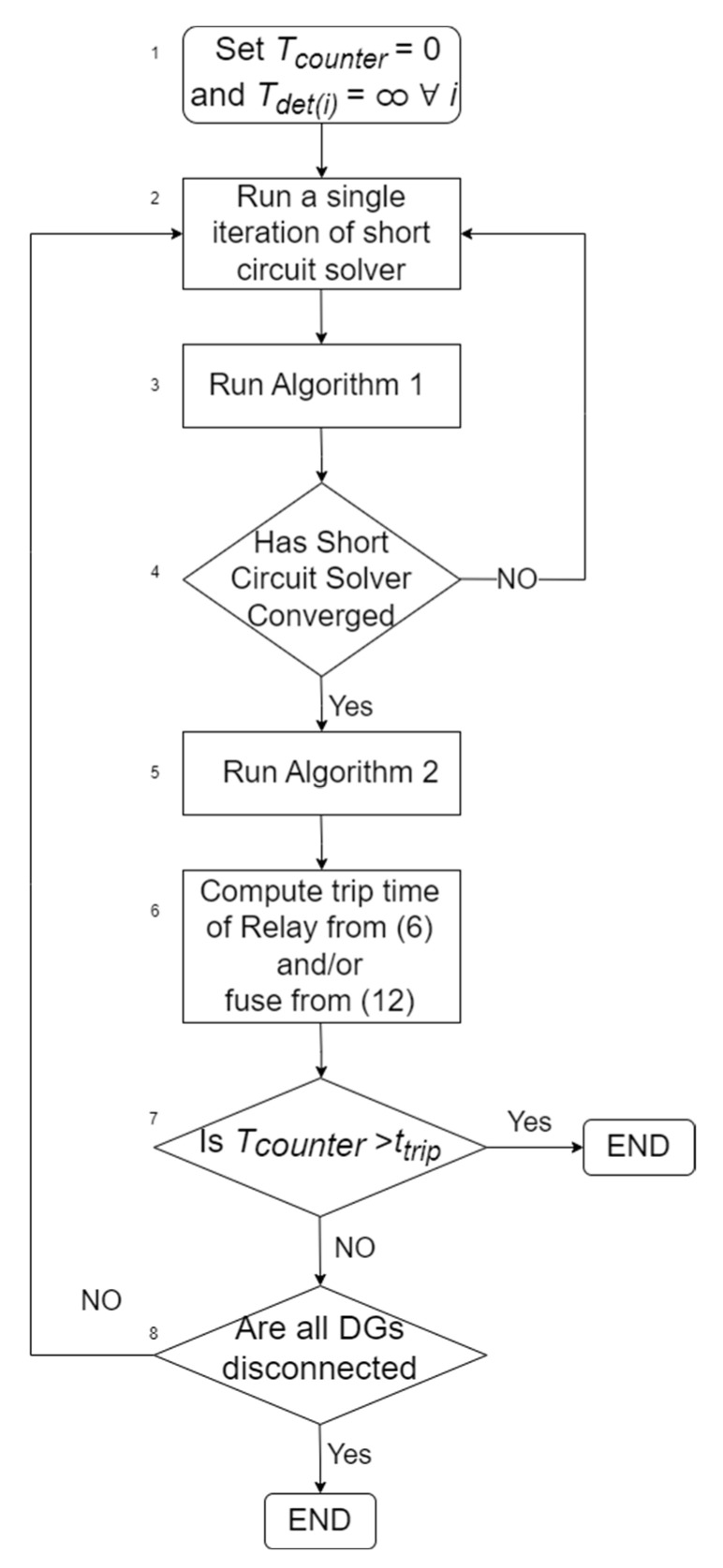
Flowchart of the proposed SCC approach.

The method proposed in this paper has been inspired by the work of Hooshyar et al. [24]. This paper contributes by improving reference [24] in the following aspects:

- Hooshyar et al. do not accurately consider modern FRT codes adopted by many countries worldwide [3,4]. For instance, although reactive power injection is required by the DGs in all the modern codes, it is ignored in [24]. On the contrary, this paper accurately considers the modern FRT codes (e.g., German code) with respect to the disconnection time and current of DGs.
- Hooshyar et al. do not clearly present the mathematical formulation of how the disconnection times and currents of DGs are incorporated into the SCC. Instead, only the implementation logic is briefly described (see Figure 5 of [24]). On the contrary, this paper proposes two algorithms (Algorithms 1 and 2 in Section 2.2) and a flowchart (Figure 5) enabling a clear mathematical incorporation of modern FRT codes into the SCC.
- Hooshyar et al. investigate only the influence of the time-varying short-circuit current on the tripping time of overcurrent relays, neglecting fuses. However, fuses constitute important parts of the protection system of distribution networks, and the precise estimation of their tripping time has great significance. This paper overcomes the previous limitation by outlining a clear model for fuses that accurately computes their burning time under time-varying fault currents (see Section 2.4).
- A case study in the IEEE 8500-node network, a real medium-voltage (MV) distribution network, is presented. Based on this, valuable conclusions are drawn regarding the influence of modern FRT codes on the tripping time of protective devices in real-world networks.

The rest of this paper is structured as follows: Section 2 describes the proposed SCC approach. Section 3 validates the proposed method against Simulink, using the IEEE 13-bus network. Section 4 presents simulation results in the IEEE 8500-node network. Finally, Section 5 presents a short discussion, while Section 6 concludes the paper.

## 2. Proposed Short-Circuit Calculation Method

In order to incorporate FRT codes into the fault analysis, three basic steps must be iteratively executed: (a) SCC using a conventional algorithm such as [12,13,14,15,16,17,18,19,20,21,22,23], (b) two algorithms computing, respectively, the injected current and disconnection time of DGs, according to the FRT standard of each country, and (c) a formula that should accurately estimate the tripping time of protective devices, accounting for the time-varying fault current. All these steps are analyzed in the next sub-sections.

### 2.1. Short-Circuit Solver

Existing SCC solvers are differentiated mainly with respect to the computation time as well as the modeling of DGs and loads [9,10,11,12,13,14,15,16,17,18,19,20,21,22,23]. In this paper, the loads and fault are modeled as constant impedances inside the ***Y_BUS_*** matrix [9,12]. However, if they can effectively incorporate DGs into their formulation, other solvers can be applied, as well (e.g., Refs. [10,11,13,14,15,16,17,18,19,20,21,22,23]). One noteworthy innovation in this paper lies in the sophisticated approach taken to model DGs within the SCC. Specifically, DGs are simulated as time-varying balanced current sources, the current and disconnection time of which are calculated by two algorithms proposed in the next sub-section, which fully represent the modern FRT standards of DGs. It is noted that some countries have opened up discussions about imposing DGs also injecting a negative-sequence current in unbalanced faults ([25], Section II.B) [26]. Nevertheless, the injection of a negative-sequence current is not studied in this paper; it is left for future research.

### 2.2. Fault Ride-Through of DGs

The ever-growing penetration of distributed generators has led many countries to revise their FRT standards so that DGs are not disconnected immediately after the fault, but remain connected for a specified period, injecting balanced reactive current [25,27]. The injected current and the time that DGs remain connected depend on the positive-sequence PCC voltage of DGs [3,4,27]. In this sub-section, two algorithms are proposed to incorporate the FRT codes into the fault analysis. Specifically, the first calculates the DG currents, and the second the disconnection order of DGs. The German FRT code [3,4] is adopted here although the algorithms present generality and can be extended to the codes of other countries, as well.

**Figure 1:**
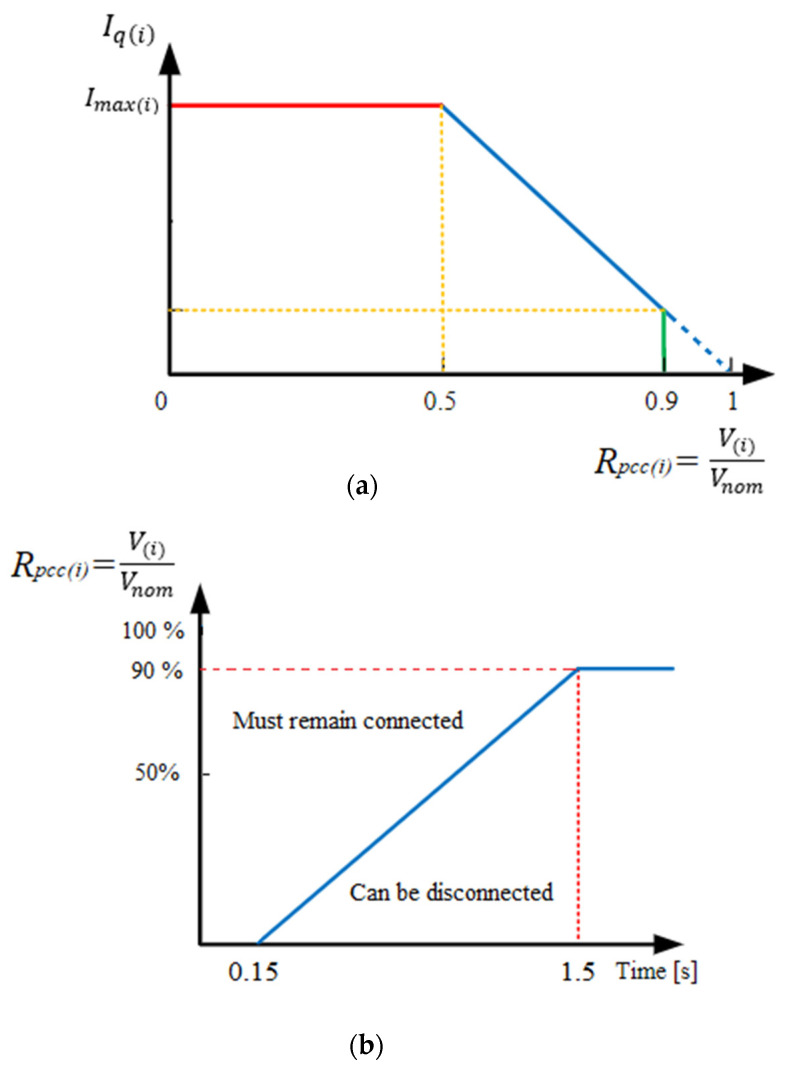
German FRT code [3,4]. From top to bottom: (**a**) required reactive current injection, (**b**) time required for DG to remain connected after the fault.

The injected positive-sequence reactive current of DGs is depicted in Figure 1a, as a function of the per unit positive-sequence PCC voltage ($R_{pcc\left(i\right)}$) [3,4]. All variables are defined in the Nomenclature section. As shown, for per unit voltages below 50%, DGs inject purely reactive current (equal to the maximum $I_{max\left(i\right)}$), while for higher voltages, DGs co-inject reactive and active current. Mathematically, the active and reactive fault current components of DG *i* are expressed by (1). The function min(*x*,*y*) outputs the minimum value between *x* and *y*.

(1a) $$I_{q\left(i\right)}=\min\left(2\cdot I_{max\left(i\right)}\cdot \left(1-R_{pcc\left(i\right)}\right),I_{max\left(i\right)}\right)\;$$

(1b) $$I_{d\left(i\right)}=\min\left(\begin{array}{cc} \frac{P_{\left(i\right)}}{3\cdot V_{\left(i\right)}}, & \sqrt{I_{max\left(i\right)}^{2}-I_{q\left(i\right)}^{2}} \end{array}\right)$$

The required FRT period of DG *i* is depicted in Figure 1b, as a function of its per unit PCC voltage, and is mathematically expressed in (2).

(2) $$\Delta t_{dis\left(i\right)}=150+\frac{1500-150}{0.9}\cdot R_{pcc\left(i\right)}\;\left(\mathrm{ms}\right)$$

As shown, the disconnection time of DGs is linearly dependent on their PCC voltage. Moreover, there is a minimum time of 150 ms that DGs should remain connected regardless of the severity of the fault. For per unit voltages above 90%, the DGs are not disconnected. Algorithms 1 and 2 below calculate, respectively, the current and disconnection order of DGs. Next to the algorithms, comments are provided to shortly explain each step. Note that the fault is assumed to be initiated always at t = 0 s (unless otherwise stated), namely, the reference system of the time starts at the fault initiation. All variables of the algorithms are defined in the Nomenclature section. The function min(***A***) outputs the minimum value of vector ***A***, while max(*x*,*y*) outputs the maximum value between *x* and *y.* Both algorithms can easily be incorporated into the fault analysis, as explained in the flowchart of Figure 5, using any conventional SCC solver [12,13,14,15,16,17,18,19,20,21,22,23].
**Algorithm 1**: Fault Current of DGsFor each connected DG i do$\;\;\;\;R_{pcc\left(i\right)}=\frac{V_{\left(i\right)}}{V_{nom}}$$\;\;\;\;If\;\;\;R_{pcc\left(i\right)}<0.9\;\;\;then\;$$\;\;\;\;\;\;\;I_{q\left(i\right)}=\min\left(2\cdot I_{max\left(i\right)}\cdot \left(1-R_{pcc\left(i\right)}\right),\;\;\;I_{max\left(i\right)}\right)$$\;\;\;\;\;\;\;I_{d\left(i\right)}=\min\left(\begin{array}{cc} \frac{P_{\left(i\right)}}{3\cdot V_{\left(i\right)}}, & \sqrt{I_{\max}^{2}-I_{q\left(i\right)}^{2}} \end{array}\right)\;$    End If   $\;\;\;\;Else\;if\;\;\;R_{pcc\left(i\right)}>0.9\;\;\;\;\;then\;$$\;\;\;\;\;\;\;I_{q\left(i\right)}=0$$\;\;\;\;\;\;\;I_{d\left(i\right)}=\frac{P_{\left(i\right)}}{3\cdot V_{\left(i\right)}}$    End IfEnd do


**Algorithm 2**: Disconnection time of DGs


For each connected DG i do



$\;\mathrm{If}\;\;R_{pcc\left(i\right)}<0.9\;\;\;and\;\;t_{\mathrm{DET}\left(i\right)}=\infty \;\;\;\;then\;$



$\;\;\;\;\;\;\;\Delta t_{dis\left(i\right)}=150+\frac{1500-150}{0.9}\cdot R_{pcc\left(i\right)}$



$\;\;\;\;\;\;\;t_{det\left(i\right)}=T_{counter}$



$\;\;\;\;\;\;\;Disconection_{time}{}_{\left(i\right)}=\Delta t_{dis\left(i\right)}+t_{det\left(i\right)}$



  End if



$\;\;Else\;if\;\;\;R_{pcc\left(i\right)}<0.9\;\;\;and\;\;t_{\det\left(i\right)}\neq \infty \;\;\;\;then\;$



$\;\;\;\;\;\;\Delta t_{dis\left(i\right)}=150+\frac{1500-150}{0.9}\cdot R_{pcc\left(i\right)}$



$\;\;\;\;\;\;Disconection_{time}{}_{\left(i\right)}=\Delta t_{dis\left(i\right)}+t_{det\left(i\right)}$



  End if



$\;\;Else\;if\;\;\;R_{pcc\left(i\right)}>0.9\;\;\;\;\;then\;$



$\;\;\;\;\;\;t_{det\left(i\right)}=\infty$



$\;\;\;\;\;\;\Delta t_{dis\left(i\right)}=\infty$



$\;\;\;\;\;\;Disconection_{time}{}_{\left(i\right)}=\infty$



  End if



End do



$\;\;\;T_{counter}=\max\left(T_{counter},\min\left(Disconection_{time}\right)\right)$



For each connected DG i do



$\;\;\;\;if\;\;T_{counter}\geq Disconection_{time}{}_{\left(i\right)}\;\;then\;$



   Disconnect DG i



End do





All the lines of both algorithms are analyzed below:

**Algorithm 1: line 1**: The algorithm is executed for all DGs connected to the grid complying with the FRT code.

**Algorithm 1: line 2**: The per unit voltage of DG i is calculated.

**Algorithm 1: lines 3–6**: If the per unit voltage of DG i falls below 0.9, the FRT of DG i is activated by injecting a current according to Equation (1).

**Algorithm 1: lines 7–10**: If the per unit voltage of DG i is above 0.9, the FRT is not active and DG i injects its nominal current.

**Algorithm 2: line 1**: The algorithm is executed for all DGs connected to the grid complying with the FRT code.

**Algorithm 2: lines 2–6**: If a fault occurs (namely $R_{pcc\left(i\right)}<0.9$) and has not yet been detected by DG i (namely $t_{det\left(i\right)}=\infty$), then its FRT period ($\Delta t_{dis\left(i\right)}$) is updated from Equation (2) and its detection time instant ($t_{det\left(i\right)}$) is set equal to $T_{counter\;.}$ As an example, if $R_{pcc\left(i\right)}$ falls below 0.9 immediately after the fault, which is usually the case, then $t_{det\left(i\right)}=0\;s$. However, there are cases of high-impedance remote faults, where $R_{pcc\left(i\right)}$ is above 0.9 for a while after the fault, and falls below it only after the voltage drop caused by the disconnection of the other DGs. In that case, $t_{det\left(i\right)}\neq 0.$ The disconnection time instant of DG i, e.g., $Disconection\_time_{\left(i\right),\;}$ is the sum of $\Delta t_{dis\left(i\right)}$ and $t_{det\left(i\right)\;}.$ For instance, if a fault is detected at 0.2 s and its FRT period is 1 s, then its $Disconection\_time_{\left(i\right)}=1.2\;s$, denoting that DG i will be disconnected at 1.2 s after the fault.

**Algorithm 2: lines 7–10**: If a fault has been already detected by DG i (namely $t_{det\left(i\right)}\neq \infty$), only the FRT period ($\Delta t_{dis\left(i\right)}$) is updated, while the detection time $t_{det\left(i\right)}$ keeps its previous value. Inevitably, $\Delta t_{dis\left(i\right)}$ varies after the disconnection of the other DGs due to the variation in network voltages. On the opposite case, $t_{det\left(i\right)}$ is not varied and is reset only after the fault ceases.

**Algorithm 2: lines 11–15**: If DG i is far away from the fault, its $R_{pcc\left(i\right)}$ will may lie above 0.9, and thus its $Disconection\_time_{\left(i\right)}$ is set to infinite, which in practical terms, means no disconnection of this DG.

**Algorithm 2: line 17**: $T_{counter}$ is updated only if its value becomes lower than the minimum disconnection time of the connected DGs. Otherwise, it remains constant until all DGs with disconnection times lower than $T_{counter}$ are disconnected in the next step. For instance, assume a network with three connected DGs and disconnection times of 1.5 s, 0.7 s, and 1.4 s, respectively. If $T_{counter}=1\;s$, then it remains constant until the second DG (with 0.7 s < 1 s) is disconnected (in the next step).

**Algorithm 2: lines 18–21**: All DGs with disconnection times lower than $T_{counter}$ are disconnected. This is because $T_{counter}$ denotes the time interval from the instant of fault initiation, while $Disconection\_time_{\left(i\right)}\;\mathrm{denotes}$ the diconnection time of DG i with respect to the fault initiation. Thus, if $T_{counter}$ surpasses $Disconection\_time_{\left(i\right)}$, DG i is disconnected.

### 2.3. Tripping Time of Relays

In the absence of DGs or in case that they are immediately disconnected after the fault, the current through the overcurrent relay is constant during the whole period of the fault, and the tripping time of the relay is easily calculated by (3) as a function of the current [24]:

(3) $$t_{trip\left(j\right)}=TDS\cdot \left(\frac{A}{{\left(\frac{I_{j}}{I_{p}}\right)}^{B}-1}+\Gamma \right)$$

where TDS, A, B, Γ are constants that determine the characteristic curve of relay (e.g., inverse, extremely inverse), $I_{j}$ is the current through the relay, and $I_{p}$ is the pick-up current [28].

**Figure 2:**
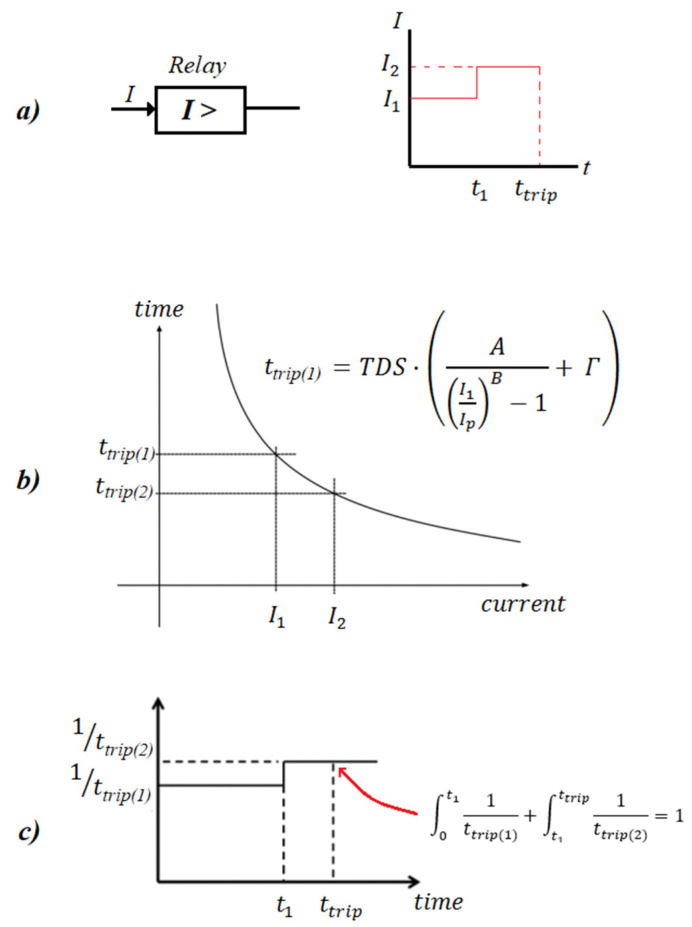
From top to bottom: (**a**) time-varying current through the relay, (**b**) characteristic curve of overcurrent relay, (**c**) integrator of digital relay.

However, modern FRT standards cause the disconnection of DGs at different time instants, thus seriously modifying the fault current flowing through the protective devices, affecting their tripping time. For instance, looking at Figure 2a, the fault currents flowing through the relay between $0\;\;\mathrm{and}\;t_{1}$ and between $t_{1}\;\mathrm{and}\;t_{trip}$ are $I{\;}_{1}\;$ and $I_{2}$, respectively, and according to Figure 2b, the two currents result in different tripping times $(t_{trip\left(1\right)}$,$\;t_{trip\left(2\right)})$. The digital relay integrates $\frac{1}{t_{trip\left(1\right)}}\;\;$ and $\;\frac{1}{t_{trip\left(2\right)}}$, according to (4), and trips the interrupting device as soon as the integrator reaches 1 (see Figure 2c).

(4) $$\int_{0}^{t_{1}}\frac{1}{t_{trip\left(1\right)}}+\int_{t_{1}}^{t_{trip}}\frac{1}{t_{trip\left(2\right)}}=1$$

Solving (4), the total tripping time $t_{trip}$ is calculated, as shown in (5) [24], as a function of the time instant that the current changes ($t_{1}$) and the tripping times $t_{trip\left(1\right)},\;t_{trip\left(2\right)}.$

(5) $$\frac{t_{1}}{t_{trip\left(1\right)}}+\frac{t_{trip}-t_{1}}{t_{trip\left(2\right)}}=1\Rightarrow t_{trip}=t_{1}+t_{trip\left(2\right)}-t_{1}\cdot \frac{t_{trip\left(2\right)}}{t_{trip\left(1\right)}}$$

Generalizing Equation (5), the tripping time of the relay for *n* current changes (from $I_{1}$ to $I_{n}$) is calculated as shown in (6):

(6) $$t_{trip}=t_{n-1}+t_{trip\left(n\right)}-\overset{n-1}{\underset{k=1}{\sum }}\left(t_{trip\left(n\right)}\cdot \frac{t_{k}-t_{k-1}}{t_{trip\left(k\right)}}\right)$$

where $t_{trip}$ is the total time required for the relay to trip, $t_{trip\left(n\right)}$ is the tripping time for current $I_{n}\;,\;\mathrm{and}\;t_{n}$ indicates the time instant of the $n^{th}$ change in the current.

### 2.4. Tripping Time of Fuses

Fuses are widely applied in distribution networks due to their simplicity and low cost, for protecting overhead lines, cables, transformers, motors, capacitor banks, etc. [29]. Under normal operation, the fuse has a negligible physical resistance, which does not affect the power system. When a fault occurs, the rise in temperature caused by the high current melts the fusible element and interrupts the fault [30]. The interruption of the fault is carried out in two stages [31,32,33,34]: (a) pre-arcing (melting) period, and (b) arcing period. The sum of these two periods yields the total clearing time $\left(t_{tct}\right)$ of the fuse.

**Figure 3:**
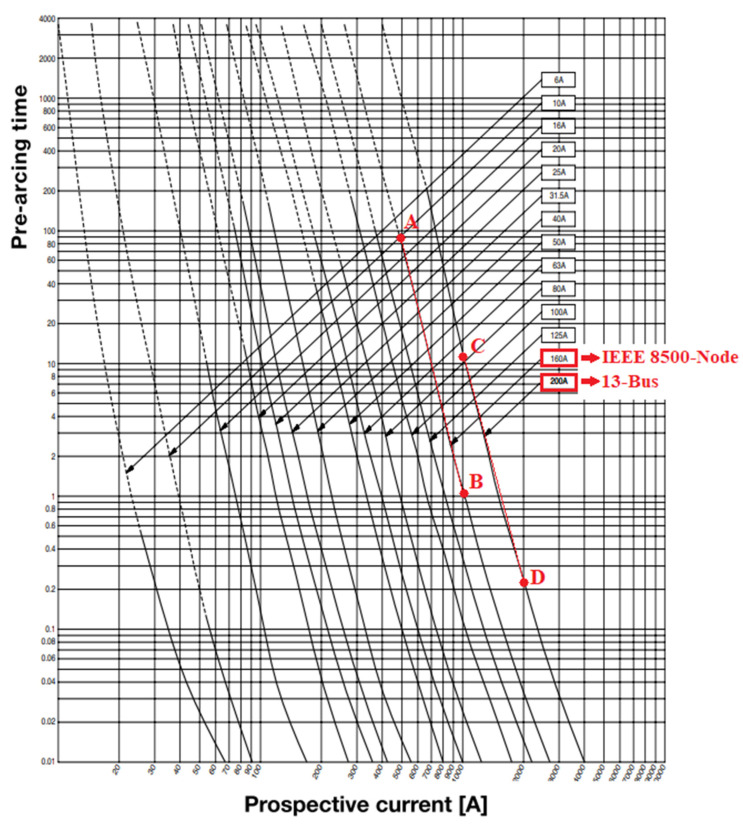
Pre-arcing time–current characteristic of CEF 6/12 kV fuses (ABB) with a current rating 6 A-200 A [35].

The pre-arcing denotes the period from the fault initiation to the fusible element melting. This period depends greatly on the fault current and is given by the manufacturer, in the form of time–current curves, usually with a logarithmic scale. As an example, the pre-arcing time–current characteristics of CEF 6/12 kV fuses (ABB), with current ratings 6 A–200 A, are quoted in Figure 3 [35]. The mathematical representation of these curves is derived using fitting techniques such as linear interpolation [29], artificial neural network (ANN) [29], genetic optimization [33], etc. In this paper, we use linear interpolation between two points, such as A–B, C–D in Figure 3, to represent the curves, as follows:

(7) $$\log t_{m}=\alpha \cdot \log{Ι}_{f}+b$$

where $t_{m}$ represents the pre-arcing time of the fuse for a fault current ${Ι}_{f}$, and α and b are the parameters that have the best fit to the curve. After the melting of the fusible element, an arc is initiated between the contacts of the fuse, which typically lasts for 5–50 ms (arcing period) [31,36], until the zero crossing of the current. In this paper, the arcing period is considered 20 ms (namely 1 cycle based on [31]). Therefore, the total clearing time of the fuse is $t_{tct}=t_{m}+0.02$ s.

**Figure 4:**
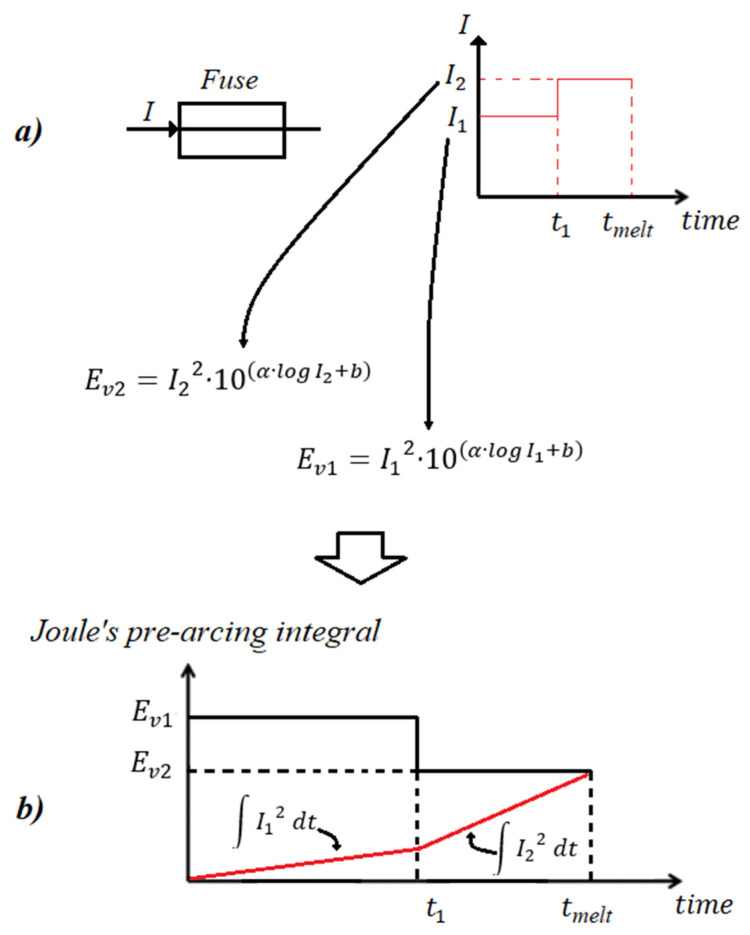
From top to bottom: (**a**) current of the fuse, changing at $t_{1}\;$ from $I_{1}$ to $I_{2}$, (**b**) Joule’s pre-arcing integral (red line) versus the time. The fuse melts at $t_{melt},$ as soon as Joule’s pre-arcing integral surpasses the virtual energy $E_{v2}$.

As already explained, traditional SCC approaches simplistically assume a constant fault current profile, and thus the estimation of melting time is directly computed from (7). Nevertheless, the estimation of melting time is complicated in cases where the fault current is time varying, as is the case in networks complying with modern FRT standards. The dynamic modeling of fuses under time-varying currents has been studied in [31,32,33,34]. In this sub-section, we adapt the methods of [31,32,33,34] to enable their integration into the steady-state SCC solvers. The authors in [31,32,33,34] argue that the fuse melts as soon as its accumulated energy (also called Joule’s pre-arcing integral) surpasses a virtual energy $\left(E_{v}\right)$, which is determined by the time–current curve of the fuse. Figure 4 depicts the methodology for computing the melting time of a fuse, assuming a time-varying fault current, from $I_{1}$ to $I_{2}$ in Figure 4a. Specifically, Joule’s pre-arcing integral is computed by (8):

(8) $$Joule^{\prime }s\;pre-arcing\;integral=\int_{0}^{t_{1}}I_{1}{}^{2}\;dt+\int_{t_{1}}^{t_{melt}}I_{2}{}^{2}\;dt=I_{1}{}^{2}\cdot t_{1}+I_{2}{}^{2}\cdot \left(t_{melt}-t_{1}\right)\;$$

where $I_{1}\;\mathrm{and}\;\;I_{2}$ are the currents via the fuse, and $t_{1}\;\;\mathrm{and}\;\;t_{melt}$ are the instant of current change and melting time, respectively.

The virtual energy (required energy for fuse to melt) depends on the current of the fuse. Specifically, in our example, the virtual energies for the currents $I_{1}\;$ and $I_{2}$ are, respectively:

(9a) $$E_{v1}=I_{1}{}^{2}\cdot 10^{\left(\alpha \cdot \log I_{1}+b\right)}\;\;$$

(9b) $$E_{v2}=I_{2}{}^{2}\cdot 10^{\left(\alpha \cdot \log I_{2}+b\right)}$$

where $E_{v1}$ is the virtual energy for current $I_{1}$ between $0\;\mathrm{and}\;t_{1}$, while $E_{v2}$ is the virtual energy for current $I_{2}$ between $t_{1}\;\mathrm{and}\;t_{melt}$. The derivation of (9) is explained in Appendix A.

As shown in Figure 4b, the fuse melts as soon as Joule’s pre-arcing integral surpasses the virtual energy. Mathematically, it is expressed as follows:

(10) $$I_{1}{}^{2}\cdot t_{1}+I_{2}{}^{2}\cdot \left(t_{melt}-t_{1}\right)=I_{2}{}^{2}\cdot 10^{\left(\alpha \cdot \log I_{2}+b\right)}\;\;\Rightarrow \;\;t_{melt}=t_{1}+\frac{I_{2}{}^{2}\cdot 10^{\left(\alpha \cdot \log I_{2}+b\right)}\;-I_{1}{}^{2}\cdot t_{1}\;}{I_{2}{}^{2}}$$

Generalizing Equation (10), the total melting time of the fuse for *n* current changes (from $I_{1}$ to $I_{n}$) is calculated according to (11):

(11) $$t_{melt}=t_{n-1}+\frac{I_{n}{}^{2}\cdot 10^{\left(\alpha \cdot \log I_{n}+b\right)}\;}{I_{n}{}^{2}}-\overset{n-1}{\underset{k=1}{\sum }}\left(I_{k}{}^{2}\cdot \frac{t_{k}-t_{k-1}}{I_{n}{}^{2}}\right)$$

where $t_{k}$ indicates the time instant of the $k^{th}$ change in the current, namely from $I_{k}$ to $I_{k+1}$. Adding the arcing period (0.02 s) to the melting time of (11), we finally take the total clearing time of the fuse:

(12) $$t_{tct}=t_{melt}+0.02\;\;\;\;\;\left(\sec\right)$$

### 2.5. Flowchart

The flowchart of the proposed SCC method is quoted in Figure 5. It consists of the following blocks:

**Block 1**: An arbitrary variable $T_{counter}$ is defined to represent the time interval from the instant of fault initiation. Assuming that the fault is initiated at 0 s, $T_{counter}$ is initially set to zero. The detection times of all DGs are set to infinite to denote that no fault is initially detected.

**Block 2**: A single iteration of a conventional SCC solver, such as [12,13,14,15,16,17,18,19,20,21,22,23], is executed.

**Block 3**: Algorithm 1 of Section 2.2 is executed, for every DG, to compute the DG currents.

**Block 4**: Blocks 2 and 3 are iteratively executed until the convergence of the SCC solver.

**Block 5**: Algorithm 2 of Section 2.2 is executed to calculate the disconnection time of DGs.

**Block 6**: The tripping time of the relay ($t_{trip}$) is computed from (6) and/or the fuse from (12). This denotes the time required for the relay/fuse to trip.

**Block 7–8**: If the time ($T_{counter}$) surpasses the tripping time of the protective device $(t_{trip},\;t_{tct}$), the protective device trips and the flowchart terminates (tripping before the last DG disconnection). Alternatively, if all DGs are disconnected, the algorithm terminates (tripping occurs after the last DG disconnection). Otherwise, it returns to block 2.

## 3. Validation of the Proposed Algorithm

**Figure 6:**
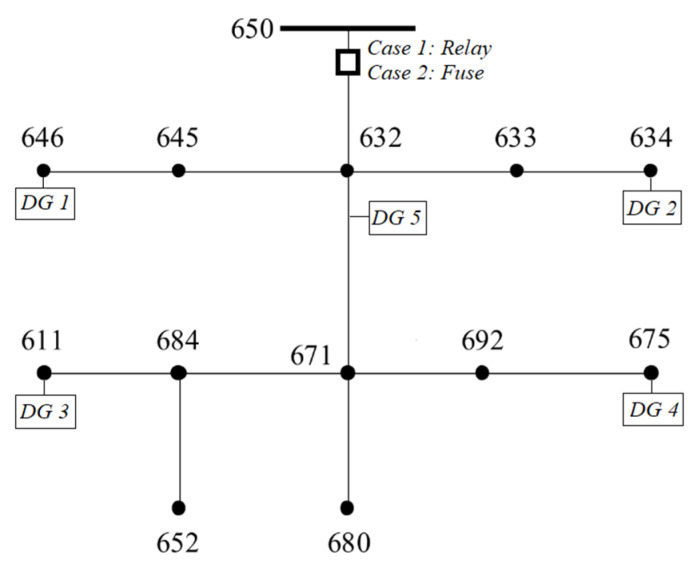
Modified IEEE 13-bus network. Two cases are examined for the substation’s protective device: case 1: relay, case 2: fuse.

In this section, the proposed algorithm is validated by comparing it against Simulink. For the validation, a modified version of the IEEE 13-bus network is used, including five DGs, as shown in Figure 6. The data of the network, DGs, and protective equipment are summarized in Table 1. For simplicity, in our analysis, the network and fault are assumed balanced, although the proposed algorithm is applicable in unbalanced networks and faults, as well. DGs follow the German FRT code [3,4] during the fault (see Figure 1). Two different protection schemes are examined: (**a**) *Case 1*: An overcurrent relay is connected at the substation (bus 650) to protect the whole network. Its characteristic curve is expressed by (3), using the parameters quoted in Table 1. (**b**) *Case 2*: A CEF 6/12 kV-200 A fuse is connected at the substation with the characteristic of Figure 3.

**Figure 7:**
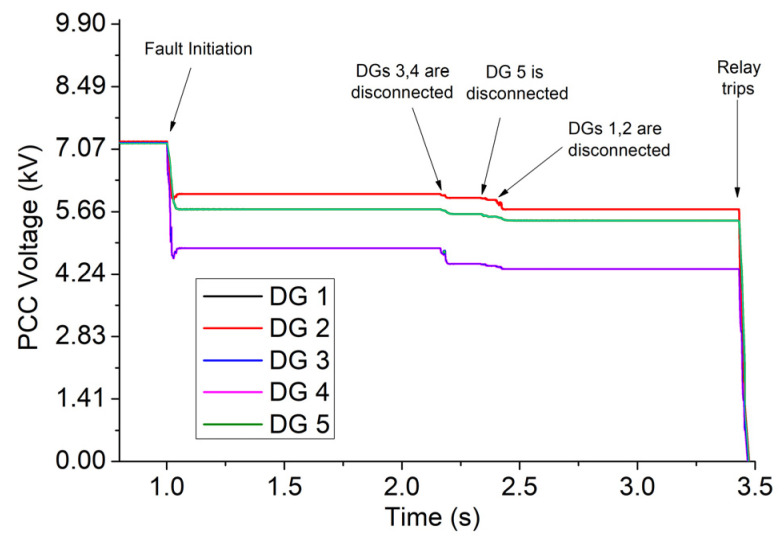
PCC voltages of DGs throughout the fault, for case 1 of a 13-bus network.

**Figure 8:**
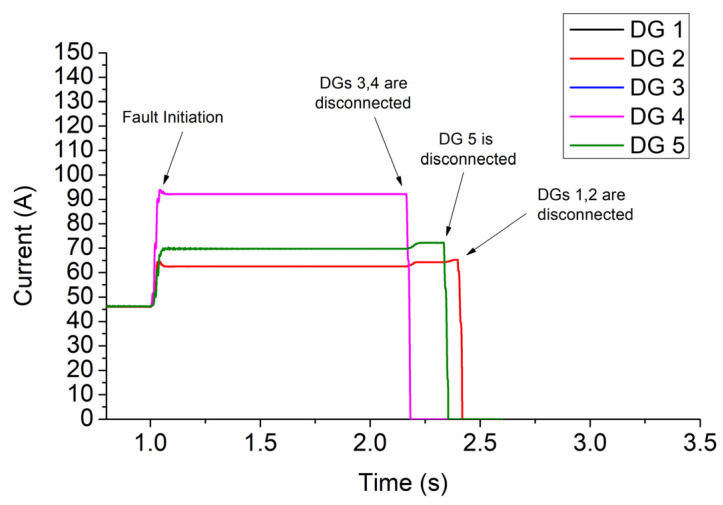
Current of DGs throughout the fault, for case 1 of a 13-bus network.

**Figure 9:**
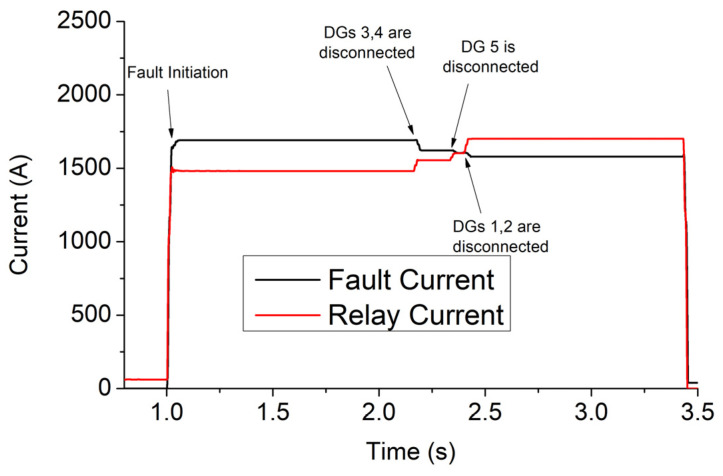
Current flowing through the fault resistance and overcurrent relay, for case 1 of a 13-bus network.

**Figure 10:**
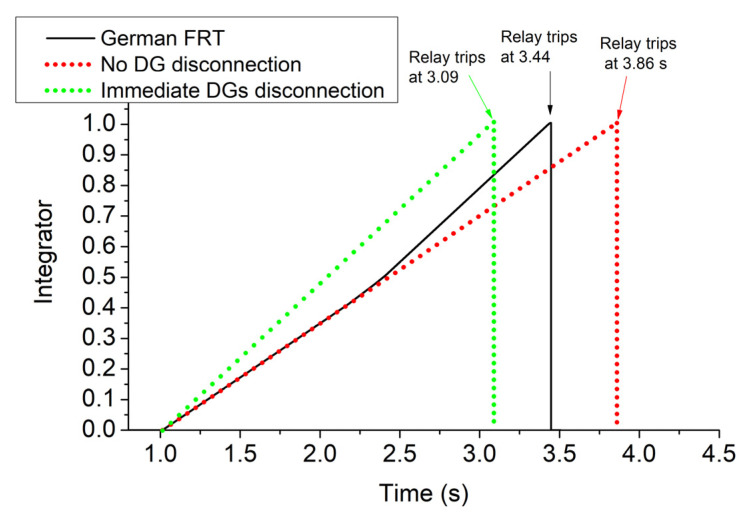
Integrator of digital relay for different FRT approaches, for case 1 of a 13-bus network. Relay trips as soon as the integrator reaches 1 [24].

A three-phase fault of 2 Ohm occurs at t = 1 s in bus 680. The results of Simulink, for case 1, are shown in Figure 7, Figure 8, Figure 9 and Figure 10. Specifically, Figure 7 depicts the PCC voltages of DGs throughout the fault. Note that DGs 1–2 as well as 3–4 have the same voltages due to the symmetry of the network, and thus their waveforms are overlapped. As shown at 1s, the voltage drops suddenly due to the fault. DGs 3 and 4 have the lowest voltage (around 4.84 kV) due to their proximity to the fault, and they are disconnected first at 2.157 s. DG 5 is disconnected at 2.318 s, while DGs 1–2 are disconnected at 2.384 s. Finally, the relay trips at 3.44 s.

The currents of DGs are illustrated in Figure 8. The highest currents are generated by DGs 3–4 due to the high voltage drop in these buses, while the lowest are generated by DGs 1–2. The levels of current flowing through the fault resistance and relay are shown in Figure 9. The former is reduced after the disconnection of DGs due to the voltage reduction, while the latter is increased thanks to the blinding that DGs cause to the relay as long as they remain connected [19]. Figure 10 depicts (with black) the value of the integrator of digital relay (see Equation (4) and Figure 2c) when the FRT of DGs complies with the German code. As shown, the rate of increase in the integrator is suddenly boosted after 2.157 s due to the increase in the relay current, which, in turn, causes a rise of $1/t_{trip\left(j\right)}$. In the same figure, for comparison, we quote the values of the integrator for two other FRT strategies: (a) all DGs remain connected throughout the whole period of fault. Practically, this consideration is adopted in [12,13,14,15,16,17,18,19,20,21,22,23]. (b) All DGs are immediately disconnected at the instant of the fault. This consideration is adopted in [9,10,11], where DGs are disregarded from the SCC. The tripping times of the relay for the three FRT strategies are 3.44 s, 3.86 s, and 3.09 s, respectively. Obviously, the FRT of DGs affects the tripping time of the relay, and therefore, the state-of-the-art SCC methods [9,10,11,12,13,14,15,16,17,18,19,20,21,22,23] may be misleading when applied to networks complying with modern FRT codes.

**Figure 11:**
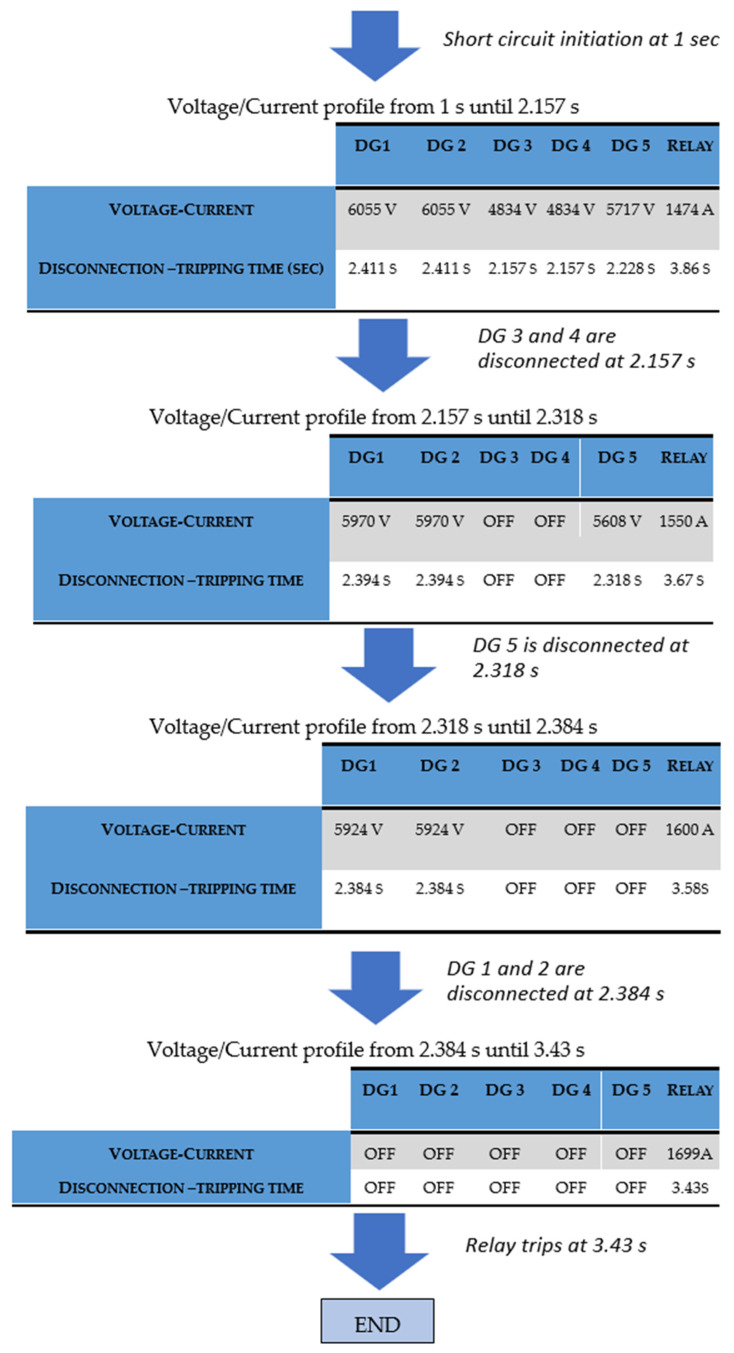
Calculation process of the proposed algorithm, for case 1 of a 13-bus network.

The calculation process of the proposed algorithm, for case 1, is shown in Figure 11, where each table represents the network’s condition at different time periods throughout the fault. Specifically, the first table shows the DG voltages and relay current when all DGs are connected (from 1 s to 2.157 s). DGs 3–4 are disconnected first at 2.157 s. The second table shows the voltages and relay current when DGs 3–4 have been disconnected (2.157 s–2.318 s). DG 5 is disconnected at 2.318 s. The third table shows the voltages and relay current when DGs 3–5 have been disconnected (2.318 s–2.384 s). DGs 1–2 are disconnected last at 2.384 s. Finally, the relay trips at 3.43 s. As shown, the results of the proposed approach (Figure 11) are in full agreement with those of Simulink (Figure 7, Figure 8, Figure 9 and Figure 10), confirming its accuracy.

Table 2 quotes the disconnection time of DGs, as well as the tripping times of the relay (case 1) and fuse (case 2), as estimated by using the proposed method and Simulink. As shown, in both cases, the results of the proposed method totally coincide with Simulink, validating its precision. Note that in case 2, the fuse trips before the disconnection of DGs 1, 2, and 5, causing a fault-induced islanding condition [37]. This is an additional benefit of the proposed SCC method, which makes the study of such adverse effects possible.

Table 3 summarizes the estimated tripping times of the relay (case 1) and fuse (case 2) for several SCC approaches and Simulink. As shown, only the proposed SCC method estimates the tripping times of relays and fuses in full agreement with Simulink. Conversely, the existing references either underestimate [9,10,11] or overestimate [12,13,14,15,16,17,18,19,20,21,22,23] the tripping time, as they overlook the time-varying fault current profile induced by the modern FRT of DGs.

## 4. Case Study in the IEEE 8500-Node Network

**Figure 12:**
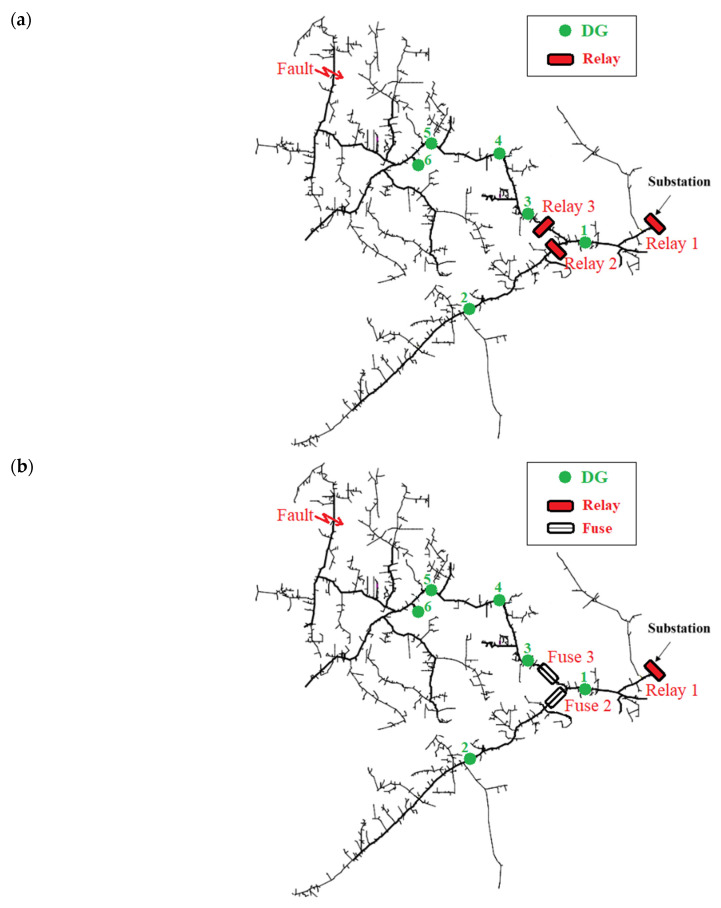
The IEEE 8500-node network. Two different protection schemes are examined: (**a**) case 1: one substation relay and two feeder relays, (**b**) case 2: one substation relay and two feeder fuses.

Simulations were carried out in the network of Figure 12, which is a slightly modified version of the IEEE 8500-node network, consisting of six DGs. It is a real North American MV distribution network [38]. Due to the large size of the network, Simulink is not applicable here. As shown in Figure 12a,b, the network is protected assuming two different protection schemes: (a) *Case 1*: One substation relay and two feeder relays (Figure 12a). (b) *Case 2*: One substation relay and two feeder fuses (Figure 12b). DG 1 is connected between the protective devices, DG 2 downstream of the relay/fuse 2, and DGs 3–6 downstream of the relay/fuse 3. All data about the network, DGs, and relays are given in Table 4. A three-phase fault with a fault resistance of 5 Ohm occurs at 0 s, at the location shown in Figure 12.

**Figure 13:**
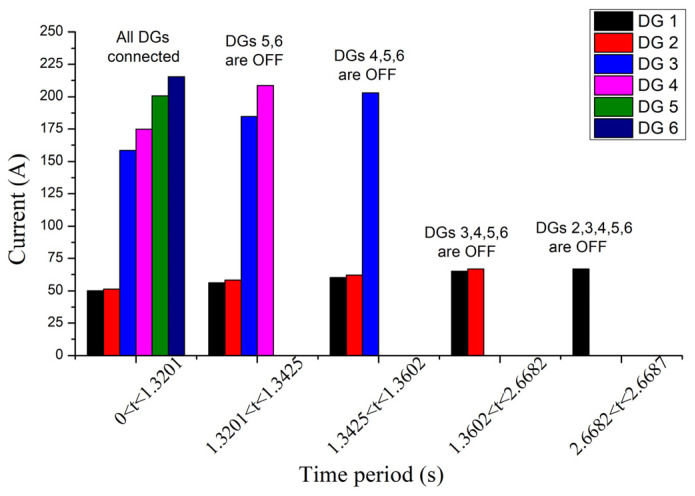
Currents of DGs in different time periods during the fault, for IEEE 8500-node network.

**Figure 14:**
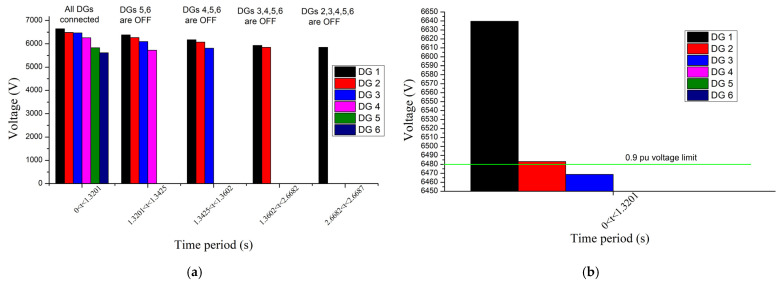
From left to right: (**a**) positive-sequence voltages of DGs in different time periods during the fault, for the IEEE 8500-node network; (**b**) zoomed diagram.

Figure 13 and Figure 14 show the magnitudes of currents and voltages of DGs, respectively. The disconnection time of DGs calculated by using the proposed approach (Algorithm 2) is depicted in Table 5. As expected, DGs that are located closer to the fault have lower voltages, higher currents, and shorter disconnection times. Moreover, the disconnection of DGs causes a voltage drop to the remaining DGs (see Figure 14), which, in turn, results in the rise of their current (see Figure 13), in accordance with (1). It can also be noted that, in our example, DGs 1 and 2 are disconnected later than the maximum FRT time of 1500 ms (see Figure 1b) because their voltage remains above 0.9 pu right after the fault (see Figure 14b) and falls below this limit only after the disconnection of DGs 5–6, namely at 1.3201 s. Thus, the FRT period of DGs 1 and 2 does not start immediately after the fault but at 1.3201 s, resulting in a delayed disconnection.

**Figure 15:**
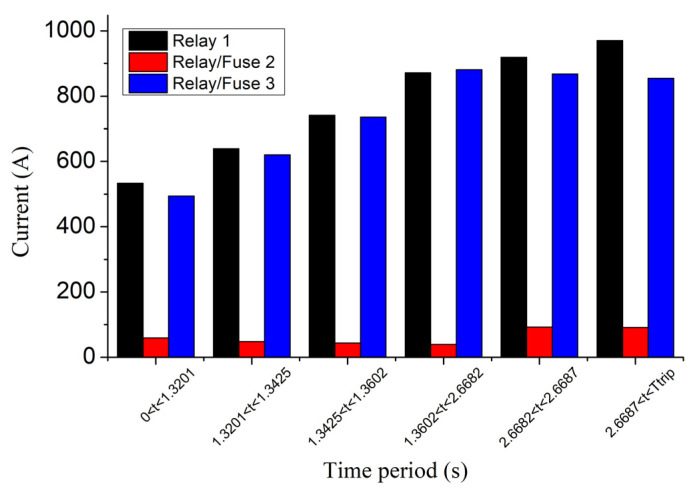
Currents of protective devices in different time periods during the fault, for the IEEE 8500-node network.

The currents flowing through the protective devices are depicted in Figure 15. Relay/fuse 2 has a low current since it is out of the fault path. The current of relay/fuse 3 is gradually increased, from 494 A to 881 A, until 2.6682 s, because of the disconnection of the downstream DGs 3–6, and subsequently decreased due to the disconnection of the upstream DGs 1 and 2. The current of relay 1 is only increased after the disconnection of DGs since they are all located downstream of the relay 1, blinding its current as long as they remain connected.

**Figure 16:**
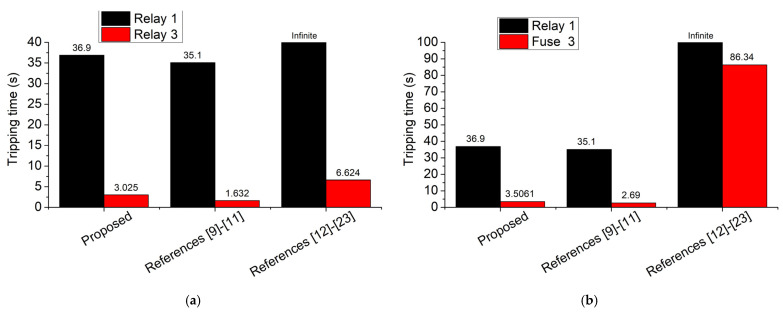
Tripping times of the different protection schemes, as they are calculated via different SCC approaches for the IEEE 8500-node network. From left to right: (**a**) case 1: see the network of Figure 12a, (**b**) case 2: see the network of Figure 12b ([9,10,11,12,13,14,15,16,17,18,19,20,21,22,23]).

The large variations in short-circuit current in Figure 15, because of the consecutive disconnections of DGs, indicate the need for a revision of the existing fault analysis methods into more contemporary approaches that will take into consideration the fluctuation of the short-circuit current. For comparison, Figure 16 illustrates the tripping times of the two different protection schemes (relays and fuse), using different SCC approaches: (a) proposed, (b) references [9,10,11], (c) references [12,13,14,15,16,17,18,19,20,21,22,23]. As shown, the three approaches output different results in both protection schemes. Specifically, for case 1, the tripping time of relay 3 is calculated by using the proposed approach, references [9,10,11], and references [12,13,14,15,16,17,18,19,20,21,22,23] as 3.025 s, 1.632 s, and 6.624 s, respectively. The tripping time of relay 1 is calculated by the three approaches 36.9 s, 35.1 s, and infinite (no tripping), respectively. For case 2, the tripping time of fuse 3 is calculated by the three approaches as 3.5061 s, 2.69 s, and 86.34 s, respectively. References [12,13,14,15,16,17,18,19,20,21,22,23] estimate a huge tripping time because most DGs are located downstream of the protective devices, blinding them throughout the whole period of the fault and thereby reducing their sensed current. References [9,10,11] estimate the lowest tripping time as the protective devices are not blinded by the DGs, which are intentionally excluded from the consideration. The proposed method computes an intermediate tripping time, as DGs are connected for a limited time, blinding the protection devices only temporarily.

Regarding the computation time, looking at the flowchart in Figure 5, the most time-consuming part is block 2, which dictates the overall computational burden of the entire flowchart. The remaining blocks exhibit a minimal computational time and are consequently disregarded. The proposed SCC method requires around 15 inner loops (from block 2 to 4) and 6 outer loops (from block 2 to 8), namely 15 × 6 = 90 executions of block 2. Since the computation time of block 2 is around 0.2 s, the total computation time is 90 × 0.2 = 18 s. This duration appears reasonable, especially considering the large scale of the network.

## 5. Discussion

With the continuous rise in the integration of DGs and the widespread adoption of modern FRT standards by numerous countries, the short-circuit current is no longer constant. Instead, it exhibits variations due to the successive disconnection of DGs. This dynamic nature of time-varying short-circuit currents introduces complexity into predicting the operating time of overcurrent relays or fuses. Conventionally, SCC methods are based on two core assumptions: immediate disconnection of DGs upon fault occurrence [9,10,11] or no disconnection of DGs [12,13,14,15,16,17,18,19,20,21,22,23]. On the other hand, this paper presents a short-circuit calculation algorithm, which accounts for the modern FRT standards. With high accuracy and low computation time, this simulates the disconnection order of DGs and the resulting time-varying short-circuit current, thus improving the precision of the estimated tripping time of protective devices.

Simulations were carried out in a 12-bus and the IEEE 8500-node network to validate the algorithm and highlight its benefits. For the IEEE 8500-node network, the assumption of “immediate DG disconnection” underestimates the tripping times of feeder relays and fuses by 1.4 s and 0.8 s, respectively. This issue arises because the assumption of immediate DG disconnection overlooks the blinding effects caused on the protective devices by the DGs as long as they remain connected. On the other hand, the assumption of “no DG disconnection” overestimates the tripping times of feeder relays and fuses by 3.6 s and 82.8 s, respectively, while it leads to the incorrect estimation of no tripping at all for the substation relay. This occurs due to the incorrect assumption that DGs continuously blind protective devices throughout the entire fault duration, without considering the sequential disconnection of DGs as mandated by FRT standards.

Lastly, it is important to note that the total harmonic distortion (THD) produced by non-linear loads can also exert an additional impact on the tripping time of protective devices, particularly when THD exceeds 10% [39]. However, utilities typically comply with established directives such as the IEEE 519-2014 standard, which imposes THD limits below 5% for distribution networks ([40]; Table 1). Consequently, this influence tends to be of minimal concern within this framework.

## 6. Conclusions

This study outlines the need for a corresponding evolution in short-circuit calculation (SCC) methods given the continuous expansion of distributed generators (DGs) complying with modern fault ride-through (FRT) standards. Specifically, advanced SCC approaches must be modified to accommodate the characteristics of contemporary FRT standards. Factors like the disconnection times of DGs, which can exert an influence on both fault currents and the tripping time of protective devices, should be integrated into the SCC for more accurate results. This paper contributes by proposing an SCC method that effectively integrates the characteristics of modern FRT standards, improving the accuracy of fault analysis, while maintaining a reasonable computation time (18 s for the IEEE 8500-node network).

## Figures and Tables

**Table 1:** Parameters of 13-bus AC network.

Nominal phase to-neutral voltage		7200 V
Resistance of the lines		0.28 Ohm/km
Self-reactance of the lines		0.33 Ohm/km
Mutual reactance of the lines		0.1 Ohm/km
Line lengths		2.5 km
Load buses		645, 633, 652, 680, 692
Constant impedance load per phase		200//800j Ohm
Nominal power of DGs		1 MW
Maximum FRT current of DGs, e.g., $I_{max\left(i\right)}$		Twice the nominal
Case 1: Relay	TDS of relay	0.5
	Peak up current of relay	600 A
	Characteristic of relay	Extremely inverse [28]
Case 2: Fuse	Model	CEF 6/12 kV
	Manufacturer	ABB
	Current rating	200 A (see Figure 3)

**Table 2:** Disconnection times of DGs and tripping times of relay (case 1) and fuse (case 2). The results of the proposed approach are with normal and Simulink with bold numbers.

Case	DGs 1 and 2	DGs 3 and 4	DG 5	Tripping Time
Case 1	2.38 s	2.16 s	2.32 s	3.43 s
	**2.38 s**	**2.16 s**	**2.32 s**	**3.44 s**
Case 2	Not disconnected	2.16 s	Not disconnected	2.27 s
	**Not disconnected**	**2.16 s**	**Not disconnected**	**2.27 s**

**Table 3:** Tripping times of relay (case 1) and fuse (case 2) for different SCC approaches.

Case	Proposed	References [12,13,14,15,16,17,18,19,20,21,22,23]	References [9,10,11]	Simulink
Case 1	3.43 s	3.86 s	3.09 s	3.44 s
Case 2	2.27 s	2.52 s	1.71 s	2.27 s

**Table 4:** Parameters of 8500-node AC network.

Nominal phase to-neutral voltage		7200 V
Resistance of the lines		Given in [38]
Self-reactance of the lines		Given in [38]
Mutual reactance of the lines		Given in [38]
Line lengths		Given in [38]
Load of each bus		Given in [38]
Total load		10.7 MW [38]
Number of DGs		6
Nominal power of DG *i* = {3, 4, 5, 6}		3 MW
Nominal power of DG *i* = {1, 2}		1 MW
Maximum FRT current of DGs, e.g., $I_{max\left(i\right)}$		Twice the nominal
FRT Code		German code [3,4]
Case 1 and Case 2	TDS of relay 1	2
	Peak up current of relay 1	600 A
	Characteristic of relay 1	Extremely inverse
Case 1: Relays	TDS of relays 2 and 3	0.4
	Peak up current of relays 2 and 3	300 A
	Characteristic of relays 2 and 3	Extremely inverse
Case 2: Fuses	Model	CEF 6/12 kV
	Manufacturer	ABB
	Current rating	160 A (see Figure 3)

**Table 5:** Disconnection times of DGs (in seconds).

DG 1	DG 2	DG 3	DG 4	DG 5	DG 6
2.6887	2.6682	1.3602	1.3425	1.3201	1.3201

## Data Availability

Not applicable.

## Nomenclature

Variable	Description	Unit
$V_{\left(i\right)}$	Positive-sequence voltage of DG *i*	V
$V_{nom}$	Nominal voltage	V
$I_{q\left(i\right)}$	Reactive current of DG *i*	A
$I_{d\left(i\right)}$	Active current of DG *i*	A
$I_{max\left(i\right)}$	Maximum FRT current of DG *i*	A
$R_{pcc\left(i\right)}$	Per unit voltage of DG *i*, e.g., Rpcci=ViVnom	pu
$P_{\left(i\right)}$	Active power of DG *i*	W
$t_{det\left(i\right)}$	Time instant of detection of the fault from DG *i.*	s
$\Delta t_{dis\left(i\right)}$	FRT period that DG *i* must remain connected, after the fault detection	s
$T_{counter}$	Counter-measuring the time interval from the instant of fault initiation	s
$t_{trip}$	Total time required for relay to trip	s
$t_{trip\left(j\right)}$	Tripping time of relay for a constant fault current $I_{j}$	s
Function	Description	Unit
min(*x*,*y*)	Outputs the minimum value between *x* and *y*	-
max(*x*,*y*)	Outputs the maximum value between *x* and *y*	-
min(***A***)	Outputs the minimum value of vector ***A***	-
Vector	Description	Unit
Disconection_time	Includes the disconnection time instants of DGs, e.g., the element $Disconection\_time_{\left(i\right)}$ is the disconnection time instant of DG *i*	s

## Appendix A

Assuming a fuse current $I_{1},\;$ the virtual energy required for the fuse to melt is given in (A1) [32]:

(A1) $$E_{v1}=I_{1}{}^{2}\cdot t_{1}$$

The time $t_{1}$ in (A1) denotes the melting time of the fuse for a current $I_{1}$ and is calculated by (7), as follows:

(A2) $$t_{1}=10^{\left(\alpha \cdot \log I_{1}+b\right)}\;$$

Combining (A1) and (A2), the virtual energy of current $I_{1}$ is given in (A3):

(A3) $$E_{v1}=I_{1}{}^{2}\cdot 10^{\left(\alpha \cdot \log I_{1}+b\right)}$$
